# Supporting Collaboration in Rehabilitation Trajectories With Information and Communication Technologies: Scoping Review

**DOI:** 10.2196/46408

**Published:** 2023-07-11

**Authors:** Jo Inge Gåsvær, Randi Jepsen, Ilona Heldal, Tobba Sudmann

**Affiliations:** 1 Faculty of Health and Social Sciences Department of Health and Function Western Norway University of Applies Sciences Bergen Norway; 2 Carasent Norge AS Dale i Sunnfjord Norway; 3 Red Cross Haugland Rehabilitation Center Flekke Norway; 4 Center for Epidemiological Research Nykøbing F Hospital Nykøbing Falster Denmark; 5 Department of Computer Science, Electrical Engineering and Mathematical Sciences Faculty of Engineering and Science Western Norway University of Applied Sciences Bergen Norway

**Keywords:** rehabilitation, shared decision-making, ICT system, decision support systems, remote dialogue, patient participation

## Abstract

**Background:**

Despite a surge in health information and communication technology (ICT), there is little evidence of lowered cost or increased quality of care. ICT may support patients, health care providers, and other stakeholders through complex rehabilitation trajectories by offering digital platforms for collaboration, shared decision-making, and safe storage of data. Yet, the questions on how ICT can become a useful tool and how the complex intersection between producers and users of ICT should be solved are challenging.

**Objective:**

This study aims to review the literature on how ICTs are used to foster collaboration among the patient, the provider, and other stakeholders.

**Methods:**

This scoping review follows the PRISMA-ScR (Preferred Reporting Items for Systematic Reviews and Meta-Analyses Extension for Scoping Reviews) guidelines. Studies were identified by searching MEDLINE (OVID), Embase (OVID), CINAHL (EBSCOhost), AMED (EBSCOhost), and Scopus. Unpublished studies were extracted from OAIster, Bielefeld Academic Search Engine, ProQuest Dissertations and Theses, NARIC, and Google Scholar. Eligible papers addressed or described a remote dialogue between stakeholders using ICT to address goals and means, provide decision support, or evaluate certain treatment modalities within a rehabilitation context. Due to the rapid development of ICTs, searches included studies published in the period of 2018-2022.

**Results:**

In total, 3206 papers (excluding duplicates) were screened. Three papers met all inclusion criteria. The papers varied in design, key findings, and key challenges. These 3 studies reported outcomes such as improvements in activity performance, participation, frequency of leaving the house, improved self-efficacy, change in patients’ perspective on possibilities, and change in professionals’ understanding of patients’ priorities. However, a misfit between the participants’ needs and the technology offered, complexity and lack of availability of the technology, difficulties with implementation and uptake, and lack of flexibility in setup and maintenance reduced the value of ICT for those involved in the studies. The low number of included papers is probably due to the complexity of remote collaboration with ICT.

**Conclusions:**

ICT has the potential to facilitate communication among stakeholders in the complex and collaborative context of rehabilitation trajectories. This scoping review indicates that there is a paucity of research considering remote ICT-supported collaboration in health care and rehabilitation trajectories. Furthermore, current ICT builds on eHealth literacy, which may differ among stakeholders, and the lack of sufficient eHealth literacy and ICT knowledge creates barriers for access to health care and rehabilitation. Lastly, the aim and results of this review are probably most relevant in high-income countries.

## Introduction

The World Health Organization (WHO) defines rehabilitation as a set of interventions designed to optimize functioning and reduce disability in individuals with health conditions in interaction with their environment. The WHO states that rehabilitation is highly person-centered, highlighting individual goal setting and preferences. WHO emphasizes the interdisciplinary workforce involved and the diversity of arenas for rehabilitation (from home or school to inpatient or outpatient hospitals) [1]. Rehabilitation is regarded as a complex and social process that requires coordinated collaboration. Information and communication technology (ICT) holds the potential for offering digital platforms for information exchange between stakeholders, collaboration, shared decision-making (SDM), and safe data storage [2].

Globally, 2.4 billion people are currently living with a health condition that could potentially be improved by rehabilitation. Due to global population growth and the rise of noncommunicable and long-lasting diseases, the estimated need for rehabilitation will gradually increase [1]. In addition, health and social care systems around the world face an increasing gap between needs and demands, a shortage of qualified staff, limited financial resources, and calls for reorganization of services [3]. Low- and high-income countries face different challenges and have to create context-specific strategies and solutions.

In high-income countries, ICT can add value to a person-centered rehabilitation process for all parties involved by using digital platforms for information exchange, collaboration, SDM, and safe data storage [2]. Despite a widespread optimism that ICT can facilitate better health and social care in terms of access, clinical outcomes, and cost-effectiveness, evidence supporting such effects is limited at best [4-7]. This may be attributed to the differences in interests and knowledge between users and producers of ICT and that some ICT solutions in health and social care processes may have none-foreseen and nonplanned effects. For example, organizational processes, roles, standards, access to information, privacy protection, and legislation may work as drivers or barriers to the implementation of ICT in health and social care services, and thus making ICT a useful tool implies considering human resources, cultures, and legal issues [8].

Barriers and drivers of access to public health care described by Levesque et al [9] illustrate the complexity of health care as a common good, as an organization, and as a personalized face-to-face service. Globally, getting access to and benefits from health and social care demands access to knowledge and resources on the individual and societal levels. The performance of health care systems is a result of the interface between the characteristics of persons, households, as well as social and physical environments, and those of health systems, organizations, and providers [9,10]. Levesque et al [9] describe dimensions and determinants of access to health care that integrate demand and supply-side factors; 5 dimensions of accessibility of services (approachability, acceptability, availability and accommodation, affordability, and appropriateness) interacting with 5 corresponding abilities of persons to generate access (ability to perceive, seek, reach, pay, and engage). There are support mechanisms and barriers in each of the phases, according to how the health care system is organized on the one side and the abilities of the patients on the other side [9]. These barriers and drivers can be used as analytical perspectives to understand why implementing ICT is complicated and expensive, and why we need to consider health and social care as interdependent [11]. ICT solutions in health care should aim at lowering barriers and facilitating drivers for access to health care and rehabilitation.

The use of ICT in health and social care services not only requires access to technology but also implicitly puts demands on the user’s eHealth literacy. Active engagement with digital services, usage of digital platforms and user interfaces, correct processing of information, engagement in own health, and preferably a feeling of safety and control are dimensions of eHealth literacy [9,12], which is affected by socioeconomic and cultural factors. Consequently, such factors should be considered in the development and implementation of ICT in health care [11]. The complexity of access to health care [9] must be considered in parallel with the complexity [12] of eHealth literacy if ICT solutions are to be useful and cost-effective.

Designing a user-friendly digital interface between patients and health care personnel is challenging, considering the diversity in eHealth literacy, access to relevant platforms, and the range of distribution in needs between staff and patients [9,12]. Among other challenges, two major problems persist: (1) integration between different ICT solutions and (2) lack of profit realization and personal satisfaction with patient-provider communication and intersection [13-15].

Patient-provider communication is a prerequisite of patient-centeredness and patient satisfaction in a variety of health care settings. Information exchange and coordination are important for the effect of treatment [16,17] and also in rehabilitation [18-25]. SDM is considered the crux of patient-centered care [26] and is dependent on information exchange and data access for everybody involved throughout the process. The SDM process is characterized by information provision and deliberation support and is a process where patients become aware of choices and their consequences. They need to understand options and have time and support to consider the most important factors for themselves. This process may require several contacts between parties, not necessarily face-to-face, including the use of decision support systems [27]. Both information provision and deliberation support can be done digitally; hence, digitalization of the SDM process in rehabilitation can be an interesting approach. eHealth literacy affects this process [12].

The complexity of patient-provider communication and collaboration, demands on eHealth literacy, and barriers and drivers for access to and usage of health care imply unresolved challenges in developing and implementing ICT to amend or support SDM processes in rehabilitation. Therefore, the aim of this review was to examine how ICT can be or become a useful tool in SDM processes in rehabilitation trajectories, where collaboration among patients, providers, and eventually others is a necessary requirement.

This paper presents and discusses findings from a scoping review of research on ICT platforms for communication, collaboration, planning, and evaluation of rehabilitation trajectories [28]. The fields of ICT, access to health care, eHealth literacy, and SDM in rehabilitation are wide ranging as fields of practice, research, and development, and a scoping review of ICT in rehabilitation trajectories is pertinent. However, acknowledging the uncertain nature of ICT development and implementation as both a resource and a challenge in literature searches and reviews opens abundant possibilities for future design thinking and action.

Rehabilitation is a contested topic where all health and social care professions have vested interests. The common denominator between rehabilitees is a need for coordinated and cross-professional assistance and services; otherwise, their needs and wants may vary greatly [29-31]. A scoping review would help us map the terrain with a broad scope, grasping patients’ and professionals’ perspectives alike.

## Methods

### Overview

The methodology for this scoping review was based on the stages in the methodological framework defined for scoping reviews [32], which has been further revised by Peters et al [33] into five phases: (1) identify aim and research questions, (2) search for relevant literature, (3) literature screening and selection, (4) data extraction, and (5) summarize and report the results. The plan for the study, including title, aim, research questions, screening process, and inclusion and exclusion criteria for the inclusion of literature, were specified in a protocol published on the internet [34]. This scoping review follows the PRISMA-ScR (Preferred Reporting Items for Systematic Reviews and Meta-Analyses Extension for Scoping Reviews) guidelines [35] (see Multimedia Appendix 1).

### Aim

The aim of this scoping review was to gain knowledge about how ICT is used to address the collaboration among the patient, provider, and other stakeholders (eg, next-of-kin, home-care services, welfare technology personnel, or landlords) through the treatment process in rehabilitation. Studies describing a remote dialogue between patients and other stakeholders using technology aiming to address SDM or goals and means, provide decision support, and evaluate treatment were of particular interest.

### Search for Relevant Literature

A search strategy was developed in collaboration with a reference librarian. Literature search strategies were developed using medical subject headings and text words related to “ICT system,” “shared decision-making,” and “rehabilitation” processes. The search strategy for MEDLINE (OVID) can be accessed digitally.

The search was initially run on February 22, 2022, and rerun on March 22, 2022. The following databases were searched: MEDLINE (OVID), Embase (OVID), CINAHL (EBSCOhost), AMED (EBSCOhost), and Scopus (see Multimedia Appendix 2). Searches were limited to studies conducted from 2018 to March 22, 2022. With the aim to locate unpublished studies, the following sources were searched: OAIster, Bielefeld Academic Search Engine, ProQuest Dissertations and Theses, NARIC, and Google Scholar. These searches were limited to studies conducted from 2018 to July 7, 2022. We limited the searches to include studies from 2018 and newer due to the fast development in this area. Furthermore, only studies in the English language were included.

### Literature Screening and Selection

Eligible papers were those which addressed or described a remote dialogue between these parties using ICT to address goals and means, provide decision support, or evaluate certain treatment modalities within a rehabilitation context. We included papers describing the development of such digital solutions, their implementations, and evaluation of the outcomes of their usage. Studies focusing on ICT solutions only for 1-way communication between patients and health care providers or papers covering only prototypes or development of some technical features without implementation in clinical work were excluded. Digital solutions in general and their influence on organizations were not of interest either. Studies about collaboration through the rehabilitation process without digital support were not included [34].

We imported identified papers into the reference manager EndNote (version 20.4.1; Clarivate Analytics) for screening of titles and abstracts to detect and eliminate duplicates. The remaining references were uploaded to Distiller SR (version 2.38; DistillerSR Inc, 2022). For further screening, 2 authors (JIG and RJ) separately screened all paper titles and abstracts for inclusion against eligibility criteria (level 1 screening). Subsequently, all eligible papers underwent full-text review by 2 authors (JIG and RJ) to confirm whether the inclusion criteria were met (level 2 screening). All discrepancies between reviewer 1 and reviewer 2 were discussed among themselves and the third (IH) and fourth authors (TS) were consulted for making decisions in case of continued disagreement. All authors fully agreed upon which articles to include in the study.

### Data Extraction and Synthesis

Data extraction forms developed by the research team were used. Two of the authors extracted data independently to ensure that all relevant information was included. Data extraction was facilitated using Distiller SR. The main focus of the analysis was the collaboration among stakeholders through ICT. The information extraction included the title of the study, author, country of authors, year published, country of selected research study, the health sector studied, size and type of selection of study sample, the technology used, type of patient focus or participation, type of decision support, type of collaboration, type of digital user interface, and key findings. After level 2 screening, data extraction was completed, and 3 papers were identified. The reviewers completed the process of data synthesis, which involved identifying important findings and noting areas with gaps in knowledge. The PRISMA-ScR flowchart for the scoping review is shown in the *Results* section.

## Results

### Overview

**Figure 1:**
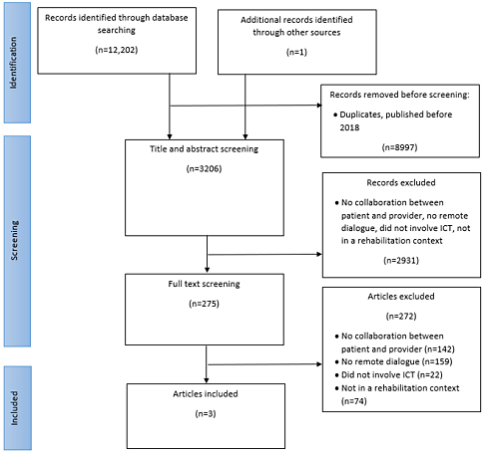
Preferred Reporting Items for Systematic Reviews and Meta-Analyses Extension for Scoping Reviews flow diagram. ICT: information and computer technology.

The literature search yielded 12,203 papers. After the removal of duplicates and papers published before 2018, a total of 3206 papers remained for screening of titles and abstracts. In this level 1 screening, many studies were excluded because they did not include the use of ICT, there was no reference to a dialogue based on shared information to address goals and means, they did not provide decision support, did not evaluate treatment, or they were not carried out in a rehabilitation setting. Much of the literature was about digital 1-way communication. It could be applications for patients’ self-monitoring (and reporting) of functioning, activity, or physical parameters. Others were about applications, text messages, or websites, which health care professionals could use to send instructions, training programs, or reminders to patients. Thus, these papers did not focus on dialogue and SDM involving both patients and practitioners. After the level 1 screening, 275 papers remained for the full-text screening, of which 3 papers met the eligibility criteria and were included in qualitative analysis. The screening process is summarized in Figure 1.

### Characteristics of Included Studies

Table 1 shows the characteristics of the 3 included studies. These 3 studies were highly different in design and technology or ICT used, sample characteristics, and key findings.

**Table 1:** Characteristics of the included studies (n=3).

Authors (year); country	Aim	Design and technology	Sample	Data collection and analysis	Type of impairment	Key findings that relate to the scoping review questions
Beit Yosef et al [36] (2022); Israel	Pilot an RCT^a^ to explore the clinical efficacy of the tele-cognitive orientation to daily occupational performance intervention for adults in the chronic phase after ABI^b^	Pragmatic exploratory partial RCT pilot study with a waitlist control crossover design examining the use of video to execute tele-cognitive orientation to daily occupational performance	16 patients, median age of 65.5 years, 3 women, 13 men, home setting. Fifteen significant others; 15 spouses; number of clinicians not given	Ratings of and scores on activity performance and participation from 3 instruments were analyzed statistically with comparisons between groups	ABI; 12 with ischemic stroke, 2 with hemorrhagic stroke, and 2 with TBI^c^	Significant improvements in activity performance on trained and untrained goals, participation reported from the participants, clinicians and significant other, and frequency of leaving the house. Partially maintained at follow-up
Strubbia et al [37] (2021); New Zealand	Describe experiences of health professionals and patients in the use of the English-language version of the iPad app aid for decision-making in occupational choice to facilitate collaborative goal setting in rehabilitation	A qualitative descriptive study using an iPad app	Eight health professionals: 7 female, 1 male participants; 6 aged 18-34 years, 2 aged ≥35 years. Eight patients: 3 female and 5 male; 6 in the age group of 18-64 years, 2 were aged ≥65 years; inpatient setting	Individual semistructured interviews conducted in person or on the internet about the understanding of aid for decision-making in occupational choice; what they liked or disliked, thoughts or feelings about using it in the clinic. Reviewed independently by 2 researchers with goal to ensure credibility, transferability, and dependability	Stroke (n=3), TBI (n=3), skin graft (n=1), chronic leg ulcer (n=1)	Changed patients’ perspective on what was possible, changed health professionals’ perspective on what was important. Facilitated shared decision-making. Lack of guidance for users. Logistical and organizational barriers, app-related and technical problems
Ali et al [38] (2021); Sweden	Evaluate the effects of person-centered care through a combined digital platform and telephone support for patients with chronic obstructive pulmonary disease and chronic heart failure	A multicenter 2-arm randomized trial examining the use of phone calls and a digital platform versus usual care	222 patients: 112 in a control group, and 110 in an intervention group. Overall, 119 were male, 103 were female; home setting	Data from questionnaires and medical records were analyzed statistically to compare groups including analyses of intention-to-treat and per-protocol	115 with COPD,^d^ 85 with CHF,^e^ 22 with both COPD and CHF	Patients who actually used the digital platform showed an improvement in general self-efficacy 3 months after the intervention. Improvements were not maintained at 6-month follow-up

^a^RCT: randomized controlled trial.

^b^ABI: acquired brain injury.

^c^TBI: traumatic brain injury.

^d^COPD: chronic obstructive pulmonary disease.

^e^CHF: chronic heart failure.

Two of the studies used quantitative methods [36,38], while the third had a qualitative approach [37]. Ali et al [38] had the highest number of participants (N=222) in their multicenter randomized trial exploring the use of a digital platform and phone calls to promote person-centered care in patients with chronic obstructive pulmonary disease and chronic heart failure. The quantitative study by Beit Yosef et al [36] piloted an RCT and included 16 patients with acquired brain injury. Occupational therapists remotely guided patients through video in the use of a global problem-solving strategy to focus on function and individual goal setting. Strubbia et al [37] included 8 health professionals and 8 patients in their interview study on the use of an iPad app to facilitate SDM with patients with stroke, traumatic brain injury, skin graft, or chronic leg ulcer.

Technology was used to address collaboration in different ways in the 3 studies. Beit Yosef et al [36] combined physical meetings and video sessions between occupational therapist and patients with acquired brain injury, with an initial meeting in person to establish a therapeutic relationship and complete a baseline assessment. Weekly video sessions over a period of 3 months were conducted before a second assessment was carried out [36]. Strubbia et al [37] also combined physical meetings and digital collaboration. In their study on health care professionals and rehabilitation patients, the professionals and patients chose up to 20 images on an iPad app representing goal topics from the activity and participation domain of the International Classification of Human Functioning, Disability and Health and rated them by importance for the patient. The patient and the health care professional then discussed the urgency of the chosen activities, and together they selected a maximum of 5 activities to focus on [37]. In contrast, Ali et al [38] only used digital collaboration in their study on patients with chronic obstructive pulmonary disease and those with chronic heart failure. In this study, the patient and the health care professional cocreated and followed up on a health plan through an optional number of phone calls. In the first telephone conversations, the health care professional established a partnership using communication skills such as listening to the patients’ narratives about daily life events and how they were affected by their condition. A health plan, including patient goals, resources, and needs, was then cocreated through discussion and agreement. A digital platform was used to support communication between phone calls, provide access to shared documentation (health plans and self-ratings), and access to reliable information sources. The digital platform was developed using participatory design including patients, patient partners, experts, and researchers [38].

The 3 studies used different measures to evaluate outcomes or experiences. Beit Yosef et al [36] used the Canadian Occupation and Performance Measure (COPM) to identify 5 functional goals and to measure activity performance in their study on patients with acquired brain injury. Activity performance was also measured through the Performance Quality Rating Scale (PQRS). Participation was measured using the Mayo-Portland Adaptability Inventory-Participation Index [36]. Strubbia et al [37] used semistructured interviews to collect and analyze health professionals’ and rehabilitation patients’ perspectives on using an iPad app for prioritizing goals. In Ali et al’s [38] study on patients with chronic obstructive pulmonary disease and patients with chronic heart failure, the primary end point was a composite score of general self-efficacy changes and hospitalization or death 6 months after randomization into usual care or the intervention group.

### Key Findings of the Included Studies

Beit Yosef et al’s [36] study with video sessions for persons with brain injuries showed significant improvements in COPM scores compared to the waitlist control group for both trained and untrained goals following the intervention. Significant improvements were also found in the PQRS and Mayo-Portland adaptability inventory-participation index scores and the patients left the house more often after the intervention. Improvements were partially maintained at follow-up. It was concluded that the intervention was feasible or effective for focusing on function and individual goal setting for adults in the chronic phase after acquired brain injury. The results gave reason to believe that strategies of problem solving learned through the intervention had a spill-over effect on other tasks. Similarly, the data from the participants’ significant other and the clinician valued the intervention as having a positive impact [36].

In the study by Strubbia et al [37] on health professionals and rehabilitation patients, the aid for decision-making in occupational choice (ADOC) app was seen as a valuable addition to the rehabilitation process by both professionals and patients because it facilitated conversations around personally meaningful goals and person-centered goal setting. The application enabled patients to understand what they could expect from the rehabilitation process and provided them access and a tool for involvement in decisions about their care. The professionals stated that the application promoted a more patient-centered approach compared to usual goal-setting practice as it gave them a better understanding of their patients’ preferences and priorities [37].

In Ali et al’s [38] study on follow-up of patients with chronic obstructive pulmonary disease and patients with the use of phone calls and a digital platform to focus on patient-centered care, no differences in composite scores were found between usual care and the intervention groups 3 and 6 months after the intervention. However, when analyzing data from participants who actually used the digital platform and the structured telephone support, there was a significant difference between groups in composite scores 3 months after the intervention but not at the 6-month follow-up [38].

### Key Challenges of the Included Studies

According to Beit Yosef et al [36], there were several methodological weaknesses in the video study with persons with brain injuries, small sample size, partial randomization, no active treatment control group, heterogeneity in goal-setting complexity between groups, wrong use of the PQRS, and nonblinded second and third assessment. The lack of improvement in general self-efficacy was assumed to be caused by the intervention’s focus on specific goals, which leads to effects on self-efficacy improvements specific to each goal. The lack of effect of the intervention on executive function in daily activities and caregiver burden was explained by the mentioned sample size and relatively high baseline scores. The authors discuss a potential ceiling effect with independent participants in need of only mild assistance in basic activities of daily living [36].

Strubbia et al [37] discuss several methodological weaknesses in their study of the ADOC application. Few participants, an exploratory study design, possible selection bias, and mandatory access to iPad to use the application were emphasized. Furthermore, the authors also discussed challenges related to implicit demands on users and the user interface with the ADOC app. Although the ADOC application was reported to be intuitive and instruction was given at the beginning of the study, both professionals and patients expressed the need for a user manual to keep up its use in clinical practice. Moreover, several logistical and organizational barriers were uncovered such as the availability of iPads in the clinic, challenges in incorporating a complex application in a pressured timetable, and lack of integration with the health care system. There were also app-related problems and technical issues with the ADOC app; a lack of possibility to create personalized goals and images in the application, no way to access a PDF treatment plan, incompatible email systems, and no print options within the organization [37].

In the study by Ali et al [38] on phone calls and a digital platform for follow-up at home, an explanation for the lack of results was a ceiling effect. The study included participants with a high initial score on general self-efficacy and feeling of disease stability, which might have reduced their need for the intervention. The effect of the intervention after 3 months was explained by the participants’ initial high degree of communication with the health care personnel and that the increase in global self-efficacy would attenuate over time independent of the intervention. The authors acknowledge that there could have been richer insights if they had explored the motivation and development of disease or rehabilitation needs through the project period. The authors also consider the need for tailoring interventions to the wants and needs of the user, to identify those persons who would benefit the most from it. The study concluded that person-centered care implies tailoring digital interventions to each patient’s unique needs [38].

## Discussion

### Principal Findings

The aim of this scoping review has been to gain knowledge about how ICT is used to support collaboration between the patient, provider, and other stakeholders (eg, next-of-kin, home-care services, welfare technology personnel, or landlords) through a rehabilitation process. Our review process suggests four different strands for discussion: (1) A low number of papers that matched the inclusion criteria; (2) the studies presented in the included papers differ in research design, sample sizes, type of technology used, and how they frame and address collaboration, effects, and limitations; (3) the complexity of ICT design and implementation in health and social care is striking; (4) there is an unaddressed implicit demand for eHealth literacy and access to health and social care.

First, since information exchange and data access for all stakeholders are prerequisites for patient-centered care, 1 potential challenge is the lack of technological solutions to support data access and exchange when needed. Lack of access complicates remote dialogue and may result in a fragmented information flow between stakeholders [13]. This was reported by Strubbia et al [37] in their study of the ADOC app on health professionals and rehabilitation patients. In their study, the lack of integration with the health care system, for example, electronic health records, logistics, problems with personalization of the application, and the lack of ability to access and distribute results, were major drawbacks with the solution [37]. These challenges might have been experienced differently by the stakeholders, even though they were unanimous in their critique.

Second, even without restrictions on data and information flow, there are numerous possible pitfalls concerning digitalizing dialogue between stakeholders in rehabilitation. As illustrated by Levesque et al [9], there are many different processes or situations a potential patient has to navigate to be able to engage in a health care encounter, for example, perceive, seek, reach, pay, engage, and interact with on the institutional or professional side to gain access. For instance, the capacity to seek health care services depends on the patient’s personal and social values, culture, gender, autonomy, socioeconomic position, and living conditions. On the societal side, cultural and social factors influence the possibility for people to accept the aspects of the service (eg, the gender or social group of providers and the beliefs associated with systems of medicine) [9]. The engagement of the patient in health care may depend on the fit between services and the patient’s needs, its timeliness, the amount of care spent in assessing health problems and determining the correct treatment, and the technical and interpersonal quality of the services provided. ICT designers are embedded in their own context and have their prejudices and knowledge gaps, like all people. To avoid what has been called “Script by design” [39-41], that is, cultural stereotypes and prejudices reiterated in the technology, ICT must incorporate a vast number of personal factors concerning the patients’ access and equivalent dimensions on the provider side through health care. This complexity can be a possible explanation for the paucity of research found in this study. The 3 included studies also report several methodological challenges. In the study by Beit Yosef et al [36] on persons with brain injuries where OTs used video to remotely guide patients in the use of a global problem-solving strategy, an initially high baseline score probably explained the lack of effect in part of the intervention. The same was the case in the study by Ali et al [38], where people with chronic obstructive pulmonary disease and patients with used a digital platform and phone calls. Ali et al [38] suggest that a high initial score on general self-efficacy and feeling of disease stability reduced the need for the intervention. This highlights the complexity described above, and as Ali et al [38] concluded, “person-centered care implies tailoring digital interventions to each patient’s unique needs.” Based on the authors’ methodological critique, we would argue that there is no best fit between the participants’ needs and wants, the baseline tests, and the outcome. Given a different methodological approach, with a systematic inclusion of participants in planning and carrying through of the studies and an explicit use of patient-reported outcome measures or experiences (where COPM sits), different outcomes might have been produced.

Third, there is a need to design digital health solutions that meet people’s needs and wants, take the users’ contexts into consideration, as well as embedding a range of possibilities for adaption to impairments or disabilities [42]. User-friendly and adaptable design is important for all stakeholders, whether it be patients, providers, and other stakeholders’ needs in different contexts. Technology development has been driven by technical possibilities to a greater extent than the needs of the different stakeholders [40,41,43]. Technology can act as a barrier against access if the design does not fit the context of use [11]. Traditional design science has not recognized the role of the organizational context in the development and implementation of technology, where a suitable demonstration context is selected after building the artifact [44]. A consequence may be that the shaping of the IT artifact condones the interests, values, and assumptions of the user end of the artifact. One can assume this is another factor influencing the paucity of research on digital collaboration in rehabilitation. The 3 included studies in this review point to this. The ceiling effect, mentioned by both Beit Yosef et al [36] and Ali et al [38], indicates that interventions are not tailored to the patient’s wants and needs. Both studies explained the lack of effects caused by initial high scores on several measures. In addition, in the study by Strubbia et al [37], several conditions were reported that indicate a lack of adaptability of the technology to individual needs. Both the need for user manuals, challenges in incorporating a complex application in a pressured timetable, and lack of personalization possibilities highlights unmet individual requirements for technology [37]. The authors do not address the most obvious lack in their methodology, including those concerned to a greater extent, probably at odds with what is recognized as the best and promising practice [30,31].

Fourth, the differences in design, results, and challenges in the included studies can also be attributed to the complexity of health care and the situations where ICT is assumed to help. Video, an iPad application, a digital platform, and phone calls are technologies used in the included studies in this review. However, what technologies are eligible for addressing the patients’ and other stakeholders’ wants and needs? This can be seen in the challenges reported in the studies, with a complex fit between methodological challenges and challenges in providing individual access for patients and the match with the provision of access from the supply side. None of the included studies are addressing the implicit knowledge demand put on users, whether these are professionals, patients, or other stakeholders. ICT research in health has shown that to be able to maximize the use of ICT in this sector all users need digital skills, dexterity, cognitive capacity, user interfaces, access to support (eg, introduction, guidance, maintenance), basic knowledge about health and health and social systems, and access to safe storage. ICT for health has been driven by technology architects and commercial interests, which creates unnecessary barriers to both commercial success and access.

### Limitations

It is important to acknowledge the limitations of this scoping review, despite following the appropriate methodology. First, the strict eligibility criteria resulted in a limited number of included studies, which may have caused the omission of important information relevant to the study's objective. In addition, restricting the search to a 5-year period starting from 2018 could have hindered the identification of relevant literature. Moreover, the decision to only include papers in English may have resulted in the exclusion of important information from non-English sources.

The search strategy for this study was developed by selecting relevant subject headings, text words, and their combinations from a larger pool of potential terms. Despite careful quality assurance measures taken during this process, there is still a chance that some crucial elements may have been inadvertently excluded from the search.

However, the low number of included papers illustrates the key finding of this study. The most salient feature of the subject matter of the study is the complexity of human and technological interfaces and collaboration, which is difficult to research.

### Conclusions

The use of ICT has been proposed as a solution to address and to support the individual management of the rise in noncommunicable diseases. Furthermore, a lesson learned after the COVID-19 pandemic is that ICT can be a valuable tool for shifting the tables among the stakeholders in rehabilitation to better meet the needs and wants of rehabilitees and other stakeholders and to provide remote support and care. Despite the widespread optimism that ICT can better access, clinical outcomes, and cost-effectiveness, evidence supporting such effects is limited. Technology use is rising globally, but there is an urgent need to include consideration about global differences not only in health burdens but also in socioeconomic factors and living conditions [45,46]. A low-tech user-friendly technical solution might have a much larger global potential to aid the rehabilitees’ process, support those concerned and their families, and reduce the demands on professional staff both in low-income and high-income countries. Low-tech and intuitive user interfaces are also paying heed to the necessity for universal design, which increases access for all.

There is an inherent contradiction between the hallmark of rehabilitation; individually tailored and complex intervention; and the study design’s lack of thorough assessments of the rehabilitees’ wants and needs, and the project’s wish for homogeneity, simplicity, and standardized goals. The understanding of a rehabilitation process is always embedded in a treatment plan, and henceforth in a research design. If these perspectives are at odds, the likelihood of success is lower.

Based on this scoping review, we still argue that ICT holds the potential to facilitate communication between stakeholders in the complex and collaborative process of rehabilitation. However, a prerequisite for eliciting this potential is to systematically include those concerned in the design and implementation process and to consider simplicity, low-tech, and low cost to lower barriers for successful user experiences and outcomes.

### Potential Implication for Information and Communication Technology Design and Further Research

Critical appreciation of the 272 papers read in full text uncovered implicit biases toward end users (rehabilitees, next-of-kin, and professionals), for example, a top-down approach, fragmented approach, or high demands on eHealth literacy. This probably creates barriers for the stakeholders’ participation in rehabilitation processes. Systematic feedback to designers seems lacking. There is an urgent need for more research on how implicit biases can be uncovered and how end users’ experiences and needs can be systematically fed back to designers. Implicit bias creates barriers to a strength-based, empowering professional relationship or needs-based inclusive design [40,41]. Critical rehabilitation studies [31] and critical disabilities studies [30,47] have repeatedly shown that if rehabilitation is to be successful, persons with disabilities must be acknowledged as competent and creative actors and included in the process of technology development and implementation from the launch of the ideas to commercialization.

## Appendix

Multimedia Appendix 1 PRISMA-ScR checklist.

Multimedia Appendix 2 Detailed search strategy for each database searched.

## Abbreviations

ADOC: aid for decision-making in occupational choice
COPM: Canadian Occupation and Performance Measure
ICT: information and computer technology
PQRS: Performance Quality Rating Scale
PRISMA-ScR: Preferred Reporting Items for Systematic Reviews and Meta-Analyses Extension for Scoping Reviews
SDM: shared decision-making
WHO: World Health Organization
