# Supplementary material for: Supporting Collaboration in Rehabilitation Trajectories With Information and Communication Technologies: Scoping Review
Source: JMIR Rehabil Assist Technol. 2023 Jul 11;10:e46408. doi: 10.2196/46408 (PMC10369310; doi:10.2196/46408)
Supplement: Multimedia Appendix 2 [file rehab_v10i1e46408_app2.pdf]

Ovid MEDLINE(R) and Epub Ahead of Print, In-Process, In-Data-Review & Other Non-Indexed Citations and Daily <1946 to March 22, 2022>

1 Health Information Systems/ 1511

2 exp medical records systems, computerized/ 45938

3 Health Information Interoperability/ 225

4 Electronic Health Records/ 24693

5 telemedicine/ or telerehabilitation/ 33393

6 User-Computer Interface/ 38976

7 Mobile Applications/ 9692

8 Cell Phone/ 9529

9 ((Patient or web) adj portal*).ab,ti,kf. 2422

10 "health information system*".ab,ti,kf. 4385

11 "medical records system*".ab,ti,kf. 491

12 ((Electronic or digital) adj2 medical record*).ab,ti,kf. 22215

13 ((electronic or digital) adj2 health record*).ab,ti,kf. 23528

14 ((electronic or digital) adj2 patient record*).ab,ti,kf. 2563

15 ((electronic or digital) adj platform*).ab,ti,kf. 1112

16 telemedicine.ab,ti,kf. 19865

17 telerehabilitation.ab,ti,kf. 1353

18 telehealth.ab,ti,kf. 9809

19 "e-health*".ab,ti,kf. 3706

20 computers, handheld/ or smartphone/ or internet/ or web browser/ or social media/ 101194

21 (mhealth or m-health).ab,ti,kf. 7944

22 ((app or apps or application* or technolog*) adj2 (mobile* or device or tablet or tablets or phone* or cellphone* or smartphone* or smart-phone* or handheld* or hand-held* or digital or communication or cell-phone)).ab,ti,kf. 35442

23 ((social adj1 (app* or media* or networking)) or website* or web-site* or internet-based or webpage* or web-page or web-based).ab,ti,kf. 109354

24 Decision Making, Shared/ 1482

25 Therapeutic Alliance/ 367

26 Goals/ 18822

27 "therapeutic alliance*".ab,ti,kf. 3098

28 goal setting.ab,ti,kf. 4696

29 goals.ab,ti,kf. 120670

30 ((Treatment or therap*) adj3 (evaluat* or assess* or outcome)).ab,ti,kf. 249315

31 ((goal* or target*) adj3 (behavio* or set* or plan* or agree* or negotiat* or discuss* or propos* or prescrib* or develop* or formulat* or elaborat* or establish* or identif* or write or written or state* or specif* or construct* or manag* or direct* or orient* or attain* or achiev* or evaluat* or cent?red)).ab,ti,kf. 382521

32 decision making.ab,ti,kf. 169274

33 (Co-production or coproduction or co-producing or coproducing or co-creat* or cocreat*).ab,ti,kf. 4811

34 ((shared or share or sharing og mutual* or collaborat*) adj4 (goal* or decision*)).ti,ab,kf. 16843

35 "Physical and Rehabilitation Medicine"/ 3478

36 exp Rehabilitation/ 335822

37 Rehabilitation Centers/ 8518

38 "Recovery of Function"/ 58149

39 exp Physical Therapy Modalities/ 169448

40 (physiotherap* or physical therap* or occupational therap* or neurorehab* or rehab* or recover*).ab,ti,kf. 977986

41 1 or 2 or 3 or 4 or 5 or 6 or 7 or 8 or 9 or 10 or 11 or 12 or 13 or 14 or 15 or 16 or 17 or 18 or 19 or 20 or 21 or 22 or 23 347766

42 24 or 25 or 27 or 28 or 29 or 30 or 31 or 32 or 33 or 34 865496

43 35 or 36 or 37 or 38 or 39 or 40 1248943

44 41 and 42 and 43 2250

Embase <1974 to 2022 Week 11>

1 medical information system/ 22309

2 electronic medical record/ 66772

3 data interoperability/ 405

4 telemedicine/ or exp teleconsultation/ or telediagnosis/ or telemonitoring/ or telepsychiatry/ or telepsychology/ or telerehabilitation/ or teletherapy/ or video consultation/ 54594

5 computer interface/ 34234

6 mobile application/ or mobile health application/ 19221

7 mobile phone/ or smartphone/ 38614

8 ((patient or web) adj portal*).ab,ti,kf. 3459

9 "health information system*".ab,ti,kf. 5505

10 "medical records system*".ab,ti,kf. 955

11 ((Electronic or digital) adj2 medical record*).ab,ti,kf. 47329

12 ((electronic or digital) adj2 health record*).ab,ti,kf. 34070

13 ((electronic or digital) adj2 patient record*).ab,ti,kf. 6355

14 ((electronic or digital) adj platform*).ab,ti,kf. 1707

15 telemedicine.ab,ti,kf. 26280

16 telerehabilitation.ab,ti,kf. 1605

17 telehealth.ab,ti,kf. 12141

18 "e-health*".ab,ti,kf. 4945

19 personal digital assistant/ 1685

20 internet/ or web-based intervention/ 118041

21 web browser/ 7301

22 social media/ 34018

23 (mhealth or m-health).ab,ti,kf. 7505

24 ((app or apps or application* or technolog*) adj2 (mobile* or device or tablet or tablets or phone* or cellphone* or smartphone* or smart-phone* or handheld* or hand-held* or digital or communication or cell-phone)).ab,ti,kf. 42557

25 ((social adj1 (app* or media* or networking)) or website* or web-site* or internet-based or webpage* or web-page or web-based).ab,ti,kf. 148920

26 1 or 2 or 3 or 4 or 5 or 6 or 7 or 8 or 9 or 10 or 11 or 12 or 13 or 14 or 15 or 16 or 17 or 18 or 19 or 20 or 21 or 22 or 23 or 24 or 25 506757

27 shared decision making/ 10706

28 therapeutic alliance/ 947

29 motivation/ or extrinsic motivation/ or incentive/ or intrinsic motivation/ 115584

30 "therapeutic alliance*".ab,ti,kf. 4419

31 goal setting.ab,ti,kf. 6654

32 goals.ab,ti,kf. 163239

33 ((Treatment or therap*) adj3 (evaluat* or assess* or outcome)).ab,ti,kf. 388770

34 ((goal* or target*) adj3 (behavio* or set* or plan* or agree* or negotiat* or discuss* or propos* or prescrib* or develop* or formulat* or elaborat* or establish* or identif* or write or written or state* or specif* or construct* or manag* or direct* or orient* or attain* or achiev* or evaluat* or cent?red)).ab,ti,kf. 522614

35 decision making.ab,ti,kf. 229744

36 (co-production or coproduction or co-producing or coproducing or co-creat* or cocreat*).ti,ab,kf. 5494

37 ((shared or share or sharing or mutial* or collaborat*) adj4 (goal* or decision*)).ab,ti,kf. 24181

38 27 or 28 or 29 or 30 or 31 or 32 or 33 or 34 or 35 or 36 or 37 1321763

39 rehabilitation/ 88763

40 rehabilitation center/ 16678

41 convalescence/ 53794

42 physiotherapy/ 94793

43 (physiotherap* or physical therap* or occupational therap* or neurorehab* or rehab* or recover*).ab,ti,kf. 1282110

44 39 or 40 or 41 or 42 or 43 1356726

45 26 and 38 and 44 3029

46 limit 45 to "pubmed/medline" 595

Cinahl*

*The number of articles below is not necessarily correct. These numbers are from a search conducted 22nd of February. The same query was run on the 25th of March, and the articles from that search were transferred to Endnote. However, the exact numbers from each of the search items and the total number of articles from the search performed on the 25th of March are lost.

#	Query	Results
S28	S26 AND S27	5,476
S27	TI (rehabilitation OR telerehabilitation OR chronic) OR AB (rehabilitation OR telerehabilitation OR chronic) OR SU (rehabilitation OR telerehabilitation OR chronic) OR MW (rehabilitation OR telerehabilitation OR chronic)	525,063
S26	S12 AND S25	48,127
S25	S13 OR S14 OR S15 OR S16 OR S17 OR S18 OR S19 OR S20 OR S21 OR S22 OR S23 OR S24	940,701
S24	TI ((decision* N1 shared) OR (decision* N1 support*) OR "decision making") OR AB ((decision* N1 shared) OR (decision* N1 support*) OR "decision making") OR SU ((decision* N1 shared) OR (decision* N1 support*) OR "decision making")	170,625
S23	TI (((patient N1 orient*) OR (participant N1 orient*) or (participant N1 perspective*) OR (patient N1 perspective*) OR (patient N1 center*) OR (participant N1 centered) OR (patient N1 participat*))) OR AB (((patient N1 orient*) OR (participant N1 orient*) or (participant N1 perspective*) OR (patient N1 perspective*) OR (patient N1 center*) OR (participant N1 centered) OR (patient N1 participat*))) OR SU (((patient N1 orient*) OR (participant N1 orient*) or (participant N1 perspective*) OR (patient N1 perspective*) OR (patient N1 center*) OR (participant N1 centered) OR (patient N1 participat*)))	69,094
S22	TI ((Treatment OR therap*) N1 (evaluat* OR assess* OR outcome)) OR AB ((Treatment OR therap*) N1 (evaluat* OR assess* OR outcome)) OR SU ((Treatment OR therap*) N1 (evaluat* OR assess* OR outcome))	449,239
S21	TI ((patient N1 relation*) OR (theraputic N1 alliance*) OR (goal N1 setting) OR goals) OR AB ((patient N1 relation*) OR (theraputic N1 alliance*) OR (goal N1 setting) OR goals) OR SU ((patient N1 relation*) OR (theraputic N1 alliance*) OR (goal N1 setting) OR goals)	243,704
S20	(MH "Outcome Assessment") OR (MH "Patient-Reported Outcomes")	51,187
S19	(MH "Consent")	19,409
S18	(MH "Goals and Objectives") OR (MH "Behavioral Objectives") OR (MH "Goal Attainment") OR (MH "Goal-Setting")	22,774
S17	(MH "Goal-Setting") OR (MH "Decision Support Techniques") OR (MH "Decision Making, Patient") OR	73,082

	(MH "Decision Making, Shared") OR (MH "Decision Making, Clinical") OR (MH "Clinical Prediction Rules") OR (MH "Decision Making, Ethical") OR (MH "Decision Making, Family")	
S16	(MH "Therapeutic Alliance") OR (MH "Professional-Client Relations")	8,420
S15	(MH "Professional-Patient Relations") OR (MH "Nurse-Patient Relations") OR (MH "Physician-Patient Relations") OR (MH "Researcher-Subject Relations")	98,472
S14	(MH "Patient Centered Care")	33,375
S13	(MH "Consumer Participation")	22,419
S12	S1 OR S2 OR S3 OR S4 OR S5 OR S6 OR S7 OR S8 OR S9 OR S10 OR S11	271,224
S11	TI (telemedicine OR telerehabilitation OR telehealth OR technology OR e-health*) OR AB (telemedicine OR telerehabilitation OR telehealth OR technology OR e-health*) OR SU (telemedicine OR telerehabilitation OR telehealth OR technology OR e-health*)	204,549
S10	TI ((electronic OR digital) N1 platform*) OR AB ((electronic OR digital) N1 platform*) OR SU ((electronic OR digital) N1 platform*)	769
S9	TI ((electronic OR digital) N2 (patient N1 record*)) OR AB ((electronic OR digital) N2 (patient N1 record*)) OR SU ((electronic OR digital) N2 (patient N1 record*))	2,110
S8	TI ((electronic OR digital) N2 (health N1 record*)) OR AB ((electronic OR digital) N2 (health N1 record*)) OR SU ((electronic OR digital) N2 (health N1 record*))	36,326
S7	TI ((electronic OR digital) N2 (health N1 record*)) OR AB ((electronic OR digital) N2 (health N1 record*)) OR SU ((electronic OR digital) N2 (health N1 record*))	36,326
S6	TI ((Electronic OR digital) N2 (medical N1 record*)) OR AB ((Electronic OR digital) N2 (medical N1 record*)) OR SU ((Electronic OR digital) N2 (medical N1 record*))	11,004
S5	TI "Medical records system" OR AB "Medical records system" OR SU "Medical records system"	118
S4	TI "Health information system" OR AB "Health information system" OR SU "Health information system"	993
S3	TI ((Patient OR web) N1 portal*) OR AB ((Patient OR web) N1 portal*) OR SU ((Patient OR web) N1 portal*)	1,917
S2	(MH "Telemedicine") OR (MH "Remote Consultation") OR (MH "Telerehabilitation")	17,076
S1	(MH "Health Information Systems+")	61,420

AMED (Allied and Complementary Medicine) <1985 to March 2022>

1 medical records/ 391

2 medical informatics/ or diagnosis computer assisted/ or therapy computer assisted/ 785

3 (information adj3 interoperabilit*).ti,hw,ab. 0

4 Telemedicine/ 1077

5 diagnosis computer assisted/ 130

6 computers/ 1622

7 telephone/ 382

8 ((Patient or web) adj portal*).ti,hw,ab. 15

9 "health information system".ti,hw,ab. 3

10 "medical records system".ti,hw,ab. 1

11 ((electronic or digital) adj2 medical record*).ti,hw,ab. 111

12 ((electronic or digital) adj2 health record*).ti,hw,ab. 78

13 ((electronic or digital) adj2 patient record*).ti,hw,ab. 18

14 ((electronic or digital) adj platform*).ti,hw,ab. 4

15 telemedicine.ti,hw,ab. 1166

16 telerehabilitation.ti,hw,ab. 155

17 telehealth.ti,hw,ab. 263

18 "e-health* ".ti,hw,ab. 28

19 exp computer systems/ 5265

20 Internet/ 1305

21 (mhealth or m-health).ti,hw,ab.42

22 ((app or apps or application* or technolog*) adj2 (mobile* or device or tablet or tablets or phone* or cellphone* or smartphone* or smart-phone* or handheld* or hand-held* or digital or communication or cell-phone)).ti,hw,ab. 459

23 ((social adj1 (app* or media* or networking)) or website* or web-site* or internet-based or webpabe* or web-page or web-based).ti,hw,ab. 1345

24 1 or 2 or 3 or 4 or 5 or 6 or 7 or 8 or 9 or 10 or 11 or 12 or 13 or 14 or 15 or 16 or 17 or 18 or 19 or 20 or 21 or 22 or 23 8049

25 "therapeutic alliance* ".ti,hw,ab. 96

26 goal setting.ti,hw,ab. 377

27 goals.ti,hw,ab. 3567

28 ((treatment or therap*) adj3 (evaluat* assess* or outcome)).ti,hw,ab. 20937

29 ((goal* or target*) adj3 (behavio* or set* or plan* or agree* or negotiat* or discuss* or propos* or prescrib* or develop* or formulat* or elaborat* or establish* or identif* or write or written or state* or specif* or construct* or manag* or direct* or orient* or attain* or achiev* or evaluat* or cent?red)).ti,hw,ab.4505

30 decision making.ti,hw,ab. 4613

31 (co-production or coproduction or co-producing or coproducing or co-creat* or cocreat*).ti,hw,ab. 48

32 ((shared or share or sharing or mutual* or collaborat*) adj4 (goal* or decision*)).ti,hw,ab. 365

33 25 or 26 or 27 or 28 or 29 or 30 or 31 or 32 31036

34 Rehabilitation/ 62787

35 rehabilitation centers/ 455

36 (physiotherap* or physical therap* or occupational therap* or neurorehab* or rehab* or recover*).ti,hw,ab. 101061

37 34 or 35 or 36 101061

38 24 and 33 and 37 391

S31	S16 AND S26 AND S30	3,443
S30	S27 OR S28 OR S29	564,348
S29	TI ((physiotherap* or "physical therap*" or "occupational therap*" or neurorehab* or rehab* or recover*)) OR AB ((physiotherap* or "physical therap*" or "occupational therap*" or neurorehab* or rehab* or recover*)) OR SU ((physiotherap* or "physical therap*" or "occupational therap*" or neurorehab* or rehab* or recover*))	406,133
S28	(MH "Rehabilitation Centers+")	9,079
S27	(MH "Rehabilitation+")	307,258
S26	S17 OR S18 OR S19 OR S20 OR S21 OR S22 OR S23 OR S24 OR S25	737,710
S25	TI (((shared or share or sharing og mutual* or collaborat*) N4 (goal* or decision*))) OR AB (((shared or share or sharing og mutual* or collaborat*) N4 (goal* or decision*))) OR SU (((shared or share or sharing og mutual* or collaborat*) N4 (goal* or decision*)))	11,384
S24	TI (Co-production or coproduction or co-producing or coproducing or co-creat* or cocreat*)) OR AB (Co-production or coproduction or co-producing or coproducing or co-creat* or cocreat*)) OR SU (Co-production or coproduction or co-producing or coproducing or co-creat* or cocreat*))	2,199
S23	TI ((Decision* N shared) or "decision making") OR AB ((Decision* N shared) or "decision making") OR SU ((Decision* N shared) or "decision making")	157,577
S22	TI (((goal* or target*) N3 (behavio* or set* or plan* or agree* or negotiat* or discuss* or propos* or prescrib* or develop* or formulat* or elaborat* or establish* or identif* or write or written or state* or specif* or construct* or manag* or direct* or orient* or attain* or achiev* or evaluat* or cent?red))) OR AB (((goal* or target*) N3 (behavio* or set* or plan* or agree* or negotiat* or discuss* or propos* or prescrib* or develop* or formulat* or elaborat* or establish* or identif* or write or written or state* or specif* or construct* or manag* or direct* or orient* or attain* or achiev* or evaluat* or cent?red))) OR SU (((goal* or target*) N3 (behavio* or set* or plan* or agree* or negotiat* or discuss* or propos* or prescrib* or develop* or formulat* or elaborat* or establish* or identif* or write or written or state* or specif* or construct* or manag* or direct* or orient* or attain* or achiev* or evaluat* or cent?red)))	98,282
S21	TI (((Treatment or therap*) N3 (evaluat* or assess* or outcome))) OR AB (((Treatment or therap*) N3 (evaluat* or assess* or outcome))) OR SU (((Treatment or therap*) N3 (evaluat* or assess* or outcome)))	492,826

S20	TI ("therapeutic alliance*" or "goal setting") OR AB ("therapeutic alliance*" or "goal setting") OR SU ("therapeutic alliance*" or "goal setting")	10,592
S19	(MH "Goals and Objectives") OR (MH "Goal-Setting") OR (MH "Goal Attainment")	21,207
S18	(MH "Therapeutic Alliance")	452
S17	(MH "Decision Making, Shared")	2,582
S16	S1 OR S2 OR S3 OR S4 OR S5 OR S6 OR S7 OR S8 OR S9 OR S10 OR S11 OR S12 OR S13 OR S14 OR S15	325,004
S15	TI (((social N1 (app* or media* or networking)) or website* or web-site* or internet-based or webpage* or web-page or web-based)) OR AB (((social N1 (app* or media* or networking)) or website* or web-site* or internet-based or webpage* or web-page or web-based)) OR SU (((social N1 (app* or media* or networking)) or website* or web-site* or internet-based or webpage* or web-page or web-based))	84,850
S14	TI (((app or apps or application* or technolog*) N2 (mobile* or device or tablet or tablets or phone* or cellphone* or smartphone* or smart-phone* or handheld* or hand-held* or digital or communication or cell-phone))) OR AB (((app or apps or application* or technolog*) N2 (mobile* or device or tablet or tablets or phone* or cellphone* or smartphone* or smart-phone* or handheld* or hand-held* or digital or communication or cell-phone))) OR SU (((app or apps or application* or technolog*) N2 (mobile* or device or tablet or tablets or phone* or cellphone* or smartphone* or smart-phone* or handheld* or hand-held* or digital or communication or cell-phone)))	32,194
S13	TI (E-health* or mhealth or m-health) OR AB (E-health* or mhealth or m-health) OR SU (E-health* or mhealth or m-health)	4,092
S12	TI (telemedicine or telerehabilitation or telehealth) OR AB (telemedicine or telerehabilitation or telehealth) OR SU (telemedicine or telerehabilitation or telehealth)	30,786
S11	TI ((electronic OR digital) N platform*) OR AB ((electronic OR digital) N platform*) OR SU ((electronic OR digital) N platform*)	11
S10	TI ((Electronic OR digital) N2 ((medical or health or patient) N record*)) OR AB ((Electronic OR digital) N2 ((medical or health or patient) N record*)) OR SU ((Electronic OR digital) N2 ((medical or health or patient) N record*))	190
S9	TI Medical records system* OR AB Medical records system* OR SU Medical records system*	2,360
S8	TI ((Patient OR web) N portal*) OR AB ((Patient OR web) N portal*) OR SU ((Patient OR web) N portal*)	32
S7	(MH "Internet+")	162,603
S6	(MH "Computers, Hand-Held+")	8,080
S5	(MH "Cellular Phone+")	9,082

S4	(MH "Mobile Applications")	10,409
S3	(MH "User-Computer Interface")	11,070
S2	(MH "Telemedicine+")	17,857
S1	(MH "Health Information Systems+")	61,716

Scopus 5523

((TITLE-ABS-KEY ((patient OR web) W/1 portal*)) OR (TITLE-ABS-KEY (health AND information AND system*)) OR (TITLE-ABS-KEY ("health information system*")) OR (TITLE-ABS-KEY ("Medical records system*")) OR (TITLE-ABS-KEY ((electronic OR digital) W/2 "medical record*")) OR (TITLE-ABS-KEY ((electronic OR digital) W/2 "health record*")) OR (TITLE-ABS-KEY ((electronic OR digital) W/2 "patient record*")) OR (TITLE-ABS-KEY ((electronic OR digital) W/1 platform*)) OR (TITLE-ABS-KEY (telemedicine OR telerehabilitation OR telehealth)) OR (TITLE-ABS-KEY (e-health* OR mhealth OR m-health)) OR (TITLE-ABS-KEY (((app OR apps OR application* OR technolog*) W/2 (mobile* OR device OR tablet OR tablets OR phone* OR cellphone* OR smartphone* OR smart-phone* OR handheld* OR hand-held* OR digital OR communication OR cell-phone)))) OR (TITLE-ABS-KEY (((social W/1 (app* OR media* OR networking)) OR website* OR web-site* OR internet-based OR webpage* OR web-page OR web-based)))) AND ((TITLE-ABS-KEY ("Therapeutic alliance*")) OR (TITLE-ABS-KEY ("goal

setting")) OR (TITLE-ABS-KEY (((treatment OR therap*) W/3 (evaluat* OR assess* OR outcome)))) OR (TITLE-ABS-KEY (((goal* OR target*) W/3 (behavio* OR set* OR plan* OR agree* OR negotiat* OR discuss* OR propos* OR prescrib* OR develop* OR formulat* OR elaborat* OR establish* OR identif* OR write OR written OR state* OR specif* OR construct* OR manag* OR direct* OR orient* OR attain* OR achiev* OR evaluat* OR cent?red)))) OR (TITLE-ABS-KEY (decision* W/1 shared)) OR (TITLE-ABS-KEY ("decision making")) OR (TITLE-ABS-KEY ((co-production OR coproduction OR co-producing OR coproducing OR co-creat* OR cocreat*))) OR (TITLE-ABS-KEY (((shared OR share OR sharing OR mutual* OR collaborat*) W/4 (goal* OR decision*)))))) AND (TITLE-ABS-KEY ((physiotherap* OR "physical therap*" OR "occupational therap*" OR neurorehab* OR rehab* OR recover*))) AND (EXCLUDE (SUBJAREA , "BIOC") OR EXCLUDE (SUBJAREA , "ENVI") OR EXCLUDE (SUBJAREA , "MATH") OR EXCLUDE (SUBJAREA , "ARTS") OR EXCLUDE (SUBJAREA , "PHAR") OR EXCLUDE (SUBJAREA , "ENER") OR EXCLUDE (SUBJAREA , "AGRI") OR EXCLUDE (SUBJAREA , "EART") OR EXCLUDE (SUBJAREA , "PHYS") OR EXCLUDE (SUBJAREA , "MATE") OR EXCLUDE (SUBJAREA , "IMMU") OR EXCLUDE (SUBJAREA , "CENG") OR EXCLUDE (SUBJAREA , "DENT") OR EXCLUDE (SUBJAREA , "CHEM") OR EXCLUDE (SUBJAREA , "VETE"))
